# Report of Clinical Observations on Continued (Typhus and Typhoid) Fever

**Published:** 1850-11

**Authors:** Austin Flint

**Affiliations:** Prof. of the Principles and Practice of Medicine, and Clinical Medicine, in the University of Buffalo


### BUFFALO MEDICAL JOURNAL

AND

### MONTHLY REVIEW.

VOL. 6. NOVEMBER, 1850. NO. 6.

### ORIGINAL COMMUNICATIONS.

ART. I.—*Report of Clinical Observations on Continued (Typhus and Typhoid) Fever. Based on an Analysis of Fifty-Two Cases.* By Austin Flint, M. D., Prof. of the Principles and Practice of Medicine, and Clinical Medicine, in the University of Buffalo.

(*Continued from page* 282.)

*Sordes.* The presence of *Sordes* on the teeth, or lips, or on both, was noted in *fourteen* cases, viz., in *six* cases of *Typhoid;* (*two* cases in private, and *four* in hospital practice;) in *six* cases of *Typhus,* and in *two* cases of *doubtful* type. In *six* of these cases the deposit was confined to the teeth; in *six* it extended to the lips, and in *two* cases its situation is not mentioned. In all the cases in which it was observed on the lips, it also existed on the teeth. In *five* of the cases in which sordes was present, the disease proved fatal. In *eleven* of the cases the tongue was either dry, or dry and hard. In *thirteen* of the cases, the tongue was more or less coated; and in the remaining case the tongue could not be inspected, owing to Parotiditis. In no case in which sordes was present, could the disease be called mild, and in most of the cases, exclusive of those which proved fatal, it was severe, and attended by considerable prostration. In the other cases the grade of intensity was not far from medium; and in the latter cases the sordes was not abundant.

This symptom very rarely occurs in the early part of the febrile career. In *one* of the cases, however, it appeared, and the deposit was abundant, on the third day. At the same time that the sordes appeared on the teeth and lips, in this case, the tongue, which for the two previous days had been natural in appearance, became thickly coated. The rapidity and abundance of the deposit in this instance, show, pretty conclusively, that it involves some change in the character of the secretions of the mouth. This case was of the *Typhus* type, and ended fatally on the fourth day.

In so far as the changes in the salivary fluids giving rise to sordes are concerned, they do not belong intrinsically to the disease, or, at all events, if they uniformly take place, they are very unequal in degree, inasmuch as the deposit is observed only in a limited number of cases. Its presence is to be regarded as denoting gravity, since, in three-sevenths of the cases in which it was observed, the disease proved fatal, and in no instance in which it occurred was the disease mild, but generally it was severe. Nevertheless, it was absent in some fatal cases, and by no means uniformly present in cases which were of a severe grade. There seems to be no foundation for the idea, which has been entertained, that it is specially significant of adynamia. Nor does it indicate, more than a dark color of the coating of the tongue, a putrescent state of the fluids. Co-existing, as it does, with more or less coating of the tongue, it seems fair to infer that it proceeds from a cause common to both; and being also found generally in connection with dryness of the tongue, we may presume that it is, in a measure, owing to the mental condition which, as has been seen, induces, in part, the latter condition.

*Hæmorrhage from the Gums.* Oozing of blood from the gums occurred in *two* cases, both of the Typhoid type, one in *hospital*, and the other in *private* practice. In *one* case (private practice) the disease was mild, unattended by delirium, accompanied by mild diarrhœa, tongue thinly coated, convalescence dating from the twenty-first day. The hemorrhagic effusion occurred in the latter part of the disease, blood exuding pretty freely, so as to color constantly the saliva, and to form incrustations on the lips. In the other case the disease proved fatal on the tenth day. The hemorrhagic effusion occurred on the sixth day, forming bloody incrustations on the lips. He complained of soreness in the throat, and a sense of dryness in the mouth. Diarrhœa did not exist in this case. The tongue was coated, at times somewhat dry, and tremulous. In neither of these cases did hemorrhage occur from the bowels, or from any part except the gums.

An *herpetic eruption about the mouth* was observed in *one* case. Lest the occurrence of this symptom may suggest a suspicion that the disease was, in this case, *remitting* fever, in which herpetic eruptions in that situation are apt to occur, it may be stated that no doubt could exist as to the diagnosis, the characteristic *maculæ*, together with other distinguishing traits, being present.

*Parotiditis.* In *five* cases, the parotid gland on one, or both sides, was the seat of inflammation, proceeding to suppuration in all but one case, and in the excepted case death took place before sufficient time had elapsed for the suppurative process to be completed. In *two* of these cases the disease was *Typhoid*, in *one*, *Typhus*, and in *two* the type was *doubtful*. One of the cases occurred in *private*, and the other cases in *hospital* practice. In the *first* case in which this complication occurred, the patient entered the hospital, March 29, 1849. Prior to this case I had never met with this complication in Continued Fever. *Two* other cases occurred at the hospital in November, 1849; and the remaining hospital case was in December, 1849. The case in private practice occurred in May, 1850.

In *two* of these *five* cases the disease proved fatal.

It is due to the importance of this intercurrent affection to devote to each of these cases some special consideration. In the *first* case, the patient entered the hospital five days after taking to his bed. The parotid of the right side began to swell on the second day after his entrance. It became greatly enlarged, hard, painful, with erythema of the surface, and proceeded to suppuration, opening below the ear, and also into the *meatus auditorius*. A large quantity of pus, and sanguinolent fluid, was discharged through these two orifices. The discharge of purulent matter continued during the career of the fever, and for several weeks afterward. A portion of cellular tissue about the size of the end of a finger, sloughed, and came away through the orifice below the ear. Owing to the swelling, and the pain in moving the jaws, the patient could not, for several days, open the mouth sufficiently for the tongue to be inspected. Convalescence was dated on the fourteenth day after his entrance. This was a case of Typhus, presenting the characteristic eruption of that type. Diarrhœa, tenderness, and meteorism were not present. Passive delirium, with getting out of bed, somnolency, some subsultus, and on one day carphologia, were noted in the record of this case.

In the *second* case, it is not stated whether the patient had been confined to the bed before entering the hospital. On the *seventh* day after his entrance, swelling of the left parotid commenced. The swelling was considerable, accompanied with redness, soreness, and pain. In this, as in all the other cases, before suppuration was accomplished, the swelling was remarkably hard, or resisting to the touch. Eight days after the commencement of the swelling, fluctuation being apparent, an incision was made, and a large quantity of pus evacuated. In two or three days afterward, another and distinct collection of pus was opened. The discharge continued, but gradually diminished, and at the time of the decease, the swelling was much reduced, and the quantity of pus small. This case terminated fatally on the twenty-third day after entrance. Diarrhœa, meteorism, and tenderness were absent in this case. Delirium, and other ataxic symptoms, were not prominent. The disease did not seem to be of a severe grade of intensity, and death, apparently, was determined by the affection of the parotid. This case is included among the cases of *doubtful* type. No autopsy was had. In the *third* case, the affection of the parotid commenced four days after convalescence was pronounced. Properly the affection in this instance should be classed among the events of convalescence, or the *sequelæ.* The patient had entered the hospital twelve days before the date of convalescence. He had not taken to his bed before entering the hospital, but had been ill for about twenty days, making strong efforts to keep about, and resist yielding to the disease. The swelling, in this case was large, tender, hard, and painful, and the surface reddened, and it proceeded to suppuration. An incision was made when fluctuation was discovered, but the abscess also ulcerated and discharged through the *meatus auditorius.* Diarrhœa, meteorism, and tenderness were not present in this case. Delirium was also absent. Eryepelas of the face succeeded the parotiditis. The patient was quite well forty days after his entrance. This case, also, is embraced among the cases of doubtful type.

In the *fourth* case, swelling of the right parotid commenced on the *sixth* day after the patient entered the hospital, and *ten* days after the attack. The patient was a female. The swelling was large, somewhat livid, with redness, tenderness, and pain. On the day succeeding that on which the swelling of the *right* parotid commenced, the *left* began to enlarge, and it soon became greatly swelled, reddened, painful, and tender. The inflammation on both sides proceeded to suppuration. On the twelfth day, fluctuation being apparent in both sides, an incision was made, giving exit to a copious purulent discharge. A portion of cellular tissue protruded through the orifice on the left side, and sloughed away. There was no discharge into the *meatus,* in this case. There was no diarrhœa in this case, but moderate tenderness, and meteorism. Delirium was slight. Moderate somnolency existed. It was difficult to fix the day of convalescence. She sat up on the twenty-fourth day after her entrance, and the last record was made on the twenty-eighth day, when there was still some discharge from the abscesses. This case was of the *Typhoid* type.

In the *fifth* case, the fever had existed five days before the patient received medical attention. In this case the patient was a female. She was suffering from nursing sore mouth when attacked with fever. On the *seventh* day after the attack, the right parotid began to swell, and became greatly enlarged, hard, reddened, and painful. Death occurred on the ninth day, the affected parotid still being greatly enlarged, and resisting to the touch. This case was of the *Typhoid* type, and occurred in private practice. It was characterized by mild diarrhœa, tenderness, meteorism, passive delirium, somnolency, eventuating in coma, the patient dying in the latter state.

Parotiditis is certainly a much rarer complication of Continued Fever than would appear from the proportion of instances in which it was present in this collection of cases. It is not mentioned in the researches of Louis, nor is it alluded to in the treatise by Bartlett. In Christison's work on fever, and in Copland's dictionary, I find it referred to, as an event which occasionally occurs toward convalescence, in some cases, proving critical, and therefore desirable. These views are not sustained by the facts in the cases that have come under my observation. In all, save one, the affection occurred before convalescence was to have been expected, and in the excepted case it occurred after convalescence was declared. In all the cases it was a serious complication, not only adding to the sufferings of the patient, but increasing the severity of the disease, and, in one instance, it appeared to be the determining cause of a fatal issue.

That this affection should have occurred in *five* out of *thirty* cases coming under observation between the dates of the first and last of the cases in which this complication existed, is a remarkable fact, of which I can offer no explanation. It may possibly be suspected that a contagious influence was transmitted from patient to patient; but there is no ground for this hypothesis. The specific form of parotiditis was not prevalent at the hospital during the period these cases transpired, nor, except in one instance, were the patients brought into contact with any other patients laboring under this complication. It occurred in two patients in the same ward, successively, with a few days' interval. In the other two hospital cases, the patients were not in wards in which any case had occurred, and the case in private practice had no connection whatever with the cases at the hospital. The fact can only be considered as exemplifying what Sydenham and others have remarked—that fever, at different times and places, may be characterized by peculiar and various local tendencies, and science is no better prepared to explain their occurrence now, than at any past period in medical history.

*Nausea and Vomiting.* Nausea and vomiting occurred in a very small proportion of the cases, viz., of the cases in *private practice*, in *three;* of those in hospital, *Typhoid*, in *three* cases; *Typhus*, in *one; doubtful type, two.* The vomiting, in all these cases, was slight, and in all it occurred early in the career of the fever, as follows:—of the private cases, in *one* instance it occurred once before the case came under my observation, and may have been owing to remedies; in *another* instance remedies were rejected for the first few days; in the *third* instance slight vomiting early in the disease. Of the *hospital* cases, *Typhoid*, in *one* instance, it is noted that the patient vomited early in the disease; in *another* instance the patient vomited on the day of attack and not afterward, and in the *third* instance remedies were rejected on the first day. In the single case of *Typhus*, the patient vomited two or three times early in the disease. Of the *two* cases of *doubtful* type, in *one* the patient vomited once on the second day, and in the *other* case vomiting occurred several times during the first few days.

Vomiting occasionally occurred after the administration of antimony, but the instances in which this symptom was obviously the result of that remedy, are not included in the above. It is to be recollected that the present enumeration embraces only those instances in which vomiting occurred after the febrile career was established. This symptom occurs in a larger proportion of cases, if the access be included. It is also to be borne in mind that in a considerable number of the hospital cases the febrile career had continued for a greater or less period before the patients came under observation. In some of these cases vomiting may have occurred, and the fact not have been noted in the *previous history.*

I should state that in the great majority of cases in which the occurrence of vomiting is not noted to have occurred, no mention is made of this symptom in the histories. It is, however, quite improbable that in any of these cases it should have occurred after the patient came under observation, and not been embraced in the record. Certainly this could not have been the case if the symptom had been in any degree prominent.

In one of the cases in which vomiting occurred, the disease was fatal. The stomach, in this case, at the autopsy, was found to be normal in size, the mucous membrane of a dingy brown color, notably thickened, and somewhat softened, without redness or vascular injection. The vomiting, in this case, occurred only two or three times, and in the early part of the disease. It was not a prominent symptom. In two fatal cases in which the stomach exhibited lesions, ulcerations existing in one case, vomiting is not noted to have occurred.

The foregoing facts are chiefly important in a negative point of view, that is to say, they go to show that nausea and vomiting rarely occur in Continued Fever, and when they are present, they are not prominent as symptoms, and are confined to the early part of the febrile career. Negative results of this kind are sometimes scarcely less valuable than those which are positive. In this instance the infrequency of vomiting is important to be taken into account in the discrimination of Continued Remitting Fever, the symptom occurring much more frequently in the latter disease.

Pains in the stomach, and other gastric symptoms, inclusive of tenderness on pressure over the epigastrium, are not mentioned in any of the histories. It is possible that had attention been directed carefully to these points, something might have been observed worthy of being recorded. Any evidences of gastric disorder, however, which were prominent, would not have been likely to have escaped notice.

*Alvine Discharges.* Questions relating to the nature of the distinction between the two types of Continued Fever, (Typhus and Typhoid,) and to the practical discrimination of each from the other, involve especially morbid conditions of the intestinal tube and their symptomatical expressions. The facts, therefore, embraced under the heads which remain to be considered under this Section, are to be studied with care in each type separately, and the results compared.

*Typhoid. Cases in Private Practice.* Of the *thirteen* cases, diarrhœa was present in *twelve.* It was mild in degree, or slight, in *seven* of these cases. It was severe and persistent in but *one* case. It was confined to the early part of the career of the fever in *four* cases. It followed the operation of cathartics in *four* cases.

*Cases in Hospital.* Of the *eighteen* cases, diarrhœa was present in *nine.* It was mild, or slight in degree in all but *two* cases, and *one* of these cases was fatal. It was limited to the early part of the career of the fever in *three* cases, and in *one* of these cases continued but for a single day. It occurred in the latter part of the disease in *one* case. It continued, more or less, through the career in *five* cases.

*Typhus.* Diarrhœa followed a cathartic in the single case of Typhus in private practice.

*Hospital Cases.* Of the *twelve* cases, diarrhœa was present in *four.*

The discharges were thin, but not too frequent in *one* case not included in the above. In all the cases it was very mild, being present but a single day in *one* case. It was limited to the early part of the career of the fever in *three* cases. It occurred in the latter part of the career in *one* case.

Adding the cases in private practice to those in hospital, diarrhœa was present in *twenty-one* of *thirty-one* cases of *Typhoid;* and in *five* of *thirteen* cases of *Typhus.* Moreover, in degree it was more uniformly slight in the cases of *Typhus.* This comparison, then, goes to show that diarrhœa occurs more frequently in the *Typhoid* than in the *Typhus* type of fever, and that, although mild in degree in the great majority of cases of both types, it is more uniformly slight in Typhus.

I should define the sense in which I have used the term *Diarrhœa.* I have not applied the term to express the character of the dejections, so much as their frequency. The character of the dejections, as will be seen in an appropriate connection, were in a large proportion of cases not ascertained. They may have been thin in some cases in which their frequency was not much, if at all increased, and in these instances diarrhœa would not be noted to exist. The patient was not considered to labor under diarrhœa while the dejections numbered only one, and occasionally two in the twenty-four hours. This explanation will serve, in some measure, to explain the greater frequency of this symptom in the cases analyzed by Louis, a fact which I find on reference to his work since the foregoing results were written. Louis evidently considers liquid dejections as constituting diarrhœa. Were it practicable to apply this rule to my cases, the number of instances in which this symptom was present, would, probably, be greater.

The reader will have observed that the proportion of instances of diarrhœa is greater in the cases in private practice than in those in hospital. Why is this? The only explanation which offers itself is, that, in most of the cases in private practice, cathartic, or laxative remedies were prescribed, while they were administered very rarely in the cases at the hospital. The latter fact is important to be taken into account in the consideration of the abdominal symptoms in the latter cases. It will be noticed that in four of the cases in private practice the diarrhœa followed, and apparently was occasioned by cathartic remedies. The practical considerations connected with this point will come up more appropriately under the head of the *Treatment of Continued Fever.*

With a view to the comparison of the fatal cases with the average of those not fatal, as respects this symptom, I will examine the former to ascertain in how many instances diarrhœa was present. Of the *two* cases of *Typhoid*, and the single case of *doubtful* type, in *private practice*, proving fatal, diarrhœa was present in all. It was a prominent and persistent symptom in *one* case; moderate, and easily relieved in *one* case, and the degree is not stated in the remaining case. It was present, after the operation of a cathartic, in the case of *Typhus* in this group. Of the *four* fatal cases of *Typhoid*, of those in *hospital*, diarrhœa was present in *two*. In *one* of these cases, it existed early in the disease, and was followed by obstinate constipation, resisting several drops of Croton oil. In another of these cases costiveness existed.* In *one* case only was the diarrhœa considerable and troublesome. Of the *two* fatal cases of *Typhus*, in *hospital*, diarrhœa was not present in either case. The dejections were *thin* in one of these cases, but not too frequent.

The connection of this symptom with lesions of the intestinal tube is an interesting point of inquiry, but this will be more appropriately considered in connection with the latter subject. *Costiveness*, more or less, was present in *five* cases of *Typhoid* occurring in *private* practice. In *one* of these cases bloody stools took place; in *one*, the costiveness was followed by diarrhœa on the administration of a cathartic; in *one* the costiveness followed diarrhœa present in the early part of the disease; in *one* it existed throughout the career, except that a cathartic excited hypercatharsis; in another case it continued, but cathartics occasioned profuse discharges; in another case it continued until the fourth day, and was followed by severe and persistent diarrhœa. *Costiveness* was present in *five* of the *Typhoid* cases in *hospital*. In one of these cases, it followed diarrhœa, and in another case it was followed by diarrhœa.

In *four* cases no dejection occurred for *three* days, and in *two* cases none for *four* days.

Of the *Typhus* cases in *hospital*, and in *private practice*, *costiveness* existed in *three*. In one of these cases no dejection occurred for several days, and in *two*, none for *four* days.

The character of the evacuations, as already stated, in a large proportion of cases, was not observed. This was owing to the difficulty of preserving them distinct, and retaining them until the time of the daily visit. In *two* of the cases in which the records contain information on this point, the dejections are stated to have been moulded, and natural in appearance. These cases were of the *Typhoid* type. In *six* cases of the same

* This case is reported in full under the head of Delirium.

type, the evacuations were thin and yellow, and in one case thin and of a brown color. Of the cases of *Typhus*, in a single instance only was the character of the evacuations noted, and in this case they were thin and yellow.

*Hemorrhage from the bowels* occurred in *two* cases, both of the *Typhoid* type. In both cases, the disease terminated in recovery. One of the cases occurred in *private practice*, and the other in *hospital*. In the former case, the discharge of blood occurred on two occasions, castor oil having been given a few hours previously to each time. The hemorrhage was pretty copious, and attended by considerable exhaustion, but on each occasion it speedily ceased after the administration of opium and the acetate of lead. Moderate tenderness over the abdomen existed in this case, but no tympanites. Costiveness existed throughout the disease in this case, and laxatives were not ventured upon except in the two instances referred to. In the other case, copious bloody evacuations also occurred on two occasions, which ceased speedily after the exhibition of enemas of morphia and tannin. They were attended by considerable prostration. The dejections at other times were thin and yellow. No abdominal distension or tympanites existed in this case, but extreme tenderness, especially in the right iliac region.

*The dejections were passed in bed*, more or less frequently in *six* cases of *Tyhoid*, (*two* in private practice and *four* in hospital,) and in *four* cases of *Typhus*, (all in hospital.) Of the *Typhoid* cases marked by this event, *two* were *fatal;* and in but *one* of the *fatal* cases of *Typhus* is this event stated to have occurred.

Evacuations in bed may be involuntary from paralysis or relaxation of the sphincter muscle ; they may arise from unconsciousness, an unconscious act of volition, if this expression be allowable ; or they may be due to indifference. On examining the histories of the cases in which this event is noted, I cannot discover that it is to be explained, in either instance, by the first of the causes just mentioned. It did not occur under circumstances of muscular prostration in which this explanation is admissible. In *two* instances it occurred in connection with a comatose condition, in which the second explanation would perhaps apply. In the remaining cases, it was apparently owing to mental indifference. The patients did not appear to appreciate the impropriety of the act, or have any concern for the consequences ; although they were easily roused, and manifested in their replies to questions more or less intelligence. Nor were they annoyed by the contact of excrement with their persons. In *two* of the cases the delirium was active. In none of the other cases, excluding the cases of coma, was somnolency a marked symptom, but in all more or less, delirium was present. In several of the cases, the patients passed evacuations in bed only occasionally, and in two or three instances, only once or twice during the continuance of the disease.

It was found practicable to prevent this accident many times by carefully watching patients, asking them frequently if they did not desire to evacuate the bowels, and occasionally placing them on the defecating chair, even if they expressed no disposition to have a dejection.

*Tympanites.* This symptom was considered present only in those cases in which there was obvious distension of the abdomen, as well as resonance on percussion. This signification should be defined, since it is stated that, by French writers, the term (or its synonym *meteorism*) is applied to all cases in which resonance is present, whether the abdomen appear distended or not.

*Typhoid. Cases in private practice.* In *ten* cases, nothing is stated on this point. In the remaining *ten* cases, *tympanites* existed in *three.* Of these *three* cases, it was moderate in degree in *two*, in *one* case existing only on one day, and it was extreme in *one*, which was a fatal case.

*Cases in Hospital.* The histories of all the *eighteen* cases contain information on this point. *Tympanites* was present in *twelve* cases. It was moderate in all cases save *one*, and was considerable in the latter case. *Typhus.* In the single case in *private* practice, *tympanites* was *not* present. In the *twelve hospital cases*, (the histories of all of which contain information on this point,) *tympanites* was present in *eight.* Of these cases it was moderate in degree in *three*, *slight* in *four*, and in *one* case the degree is not stated. Adding together all the cases of *Typhoid* on the one hand and those of *Typhus* on the other, tympanites thus was present in *sixteen* of the *twenty-eight* cases of the former type in which this symptom was mentioned ; and in *eight* of the *thirteen* cases of *Typhus*, proportions not very far from being equal.

As respects degree, there does not seem to be any ground for distinction between the two types. It would, perhaps, be fairer to compare the *hospital* cases of *Typhoid* and *Typhus*, excluding the cases in *private* practice, since all the cases in the latter group, save two, were of the former type. In this view, tympanites occurred in a ratio exactly equal in the two types, viz., 12–18 of the cases of *Typhoid*, and 8–12 of the cases of *Typhus*, i. e., in both 4–6. These results are not what I had anticipated. I had supposed this symptom was considerably more frequent in its occurrence in the cases of *Typhoid* than in those of *Typhus.*

I know not how to explain the comparative infrequency of this symptom in the cases in private practice, except by reference to the fact which was adduced in explanation of the greater frequency of diarrhœa in the latter group of cases. The inquiry arises, what connection has *tympanites* with *diarrhœa?* With reference to this inquiry, I have examined the cases in which tympanites was present, in order to ascertain in how many of these cases diarrhœa co-existed. In the *four* cases in private practice in which tympanites was present, more or less diarrhœa existed in all. In the *Typhoid hospital* cases, diarrhœa co-existed with tympanites in *eight,* and tympanites was present without diarrhœa in *four.* In the *eight* cases of *Typhus* in which tympanites existed, in *all* diarrhœa did *not* co-exist. In one case it is noted that the discharges were thin and yellow, but not too frequent.

These results are curious. In so far as these cases afford data for statistical inferences, it would seem that, in the *Typhoid* type, tympanites, in the majority of cases, is accompanied by diarrhœa, but that the former is present without the latter in all cases of *Typhus !* Of course the number of observations is much too limited to authorize such an induction ; nevertheless, the contrast is too striking to be considered wholly fortuitous.

With respect to the concurrence of tympanites and diarrhœa, it should be added, what will suggest itself to the reader as a corollary of the above facts, that diarrhœa, in both *Typhoid* and *Typhus,* but (according to the above data) oftener in the latter, is present without tympanites. This symptom was present in all the *fatal* cases of *Typhoid,* save *one ;* and in *each* of the fatal cases of *Typhus,* save *one.* Of the *Typhoid* cases, it was extreme in *one* case, moderate in *two* cases, and the degree is not stated in the remaining *three* cases in which the symptom was present. Of the cases of *Typhus,* it was moderate in one case, and the degree not stated in the other case in which the symptom was present.

*Abdominal tenderness. Typhoid.* In *two* of the histories of the cases in *private* practice, there is no allusion to tenderness on pressure over the abdomen. In the remaining *eleven* (*Typhoid*) cases, more or less tenderness was present in *eight.* The tenderness was considerable in degree in *one* case, and either moderate or slight in the remainder (*seven*) cases. Of these cases, the tenderness was either confined to, or more marked in the *right iliac* region in *four* cases, and in the other cases the situation is not stated. In the *Hospital Typhoid* cases, information is contained on this point in the histories of all. Of the *eighteen* cases, more or less *tenderness* was present in *eleven.* The tenderness was either moderate or slight in all but *three* cases. It is noted as especially marked in the *right iliac* region in *two* cases, and in *both iliac regions* in *four* cases.

*Typhus.* Slight tenderness existed in the single case of *Typhus* in *private* practice. Of the Hospital cases of *Typhus,* information on this point is contained in all the histories. Of the *twelve* cases, tenderness existed in *five.* In all it was slight in degree. The situation of the tenderness is not noted except in one case. In this case, it existed around the umbilicus ; it was present at the early part of the disease, and disappeared during the febrile career. Bringing the two types into comparison as respects this symptom, it was present in *nineteen* of *twenty-eight* cases of *Typhoid,* and in *six* of *thirteen* cases of *Typhus,* showing a considerably larger proportion in the former.

In order to ascertain if there exist any constancy of connection between this symptom and that last considered, viz., tympanites, I have examined the histories of all the cases in which tenderness existed, and enumerated those in which tympanites co-existed. Tenderness with tympanites existed in *ten* cases of *Typhoid,* (*three* in private practice and *seven* in hospital,) and in *four* cases of *Typhus.* In other words, tympanites did not co-exist in *ten* of the cases of *Typhoïd* in which tenderness was present, and in *ten* of the cases of *Typhus* in which tenderness existed, tympanites was absent.

On comparing now the number of cases in which tenderness was present in both types, with the number of cases in which tympanites existed, it is obvious, not only that tenderness exists without tympanites in a certain proportion of cases, but that tympanites is present without tenderness in a certain proportion of cases. Thus there exists no relation of dependency between these two symptoms, since either may be present without the other.

It remains to inquire respecting the connection of tenderness with diarrhœa. Of the *Typhoid* cases in which tenderness was present, diarrhœa co-existed in *ten,* (*five* in private practice and *five* in hospital.) Tenderness was present without diarrhœa in *nine* cases, (*three* in private practice and *six* in hospital.) Of the cases of *Typhus,* tenderness co-existed with diarrhœa in *two.* Tenderness was present without diarrhœa in *four* cases. Diarrhœa existed without tenderness in *four* cases of *Typhoid,* and in *two* cases *of Typhus.*

These results show that the two symptoms, *tenderness* and *diarrhœa,* are not connected by any kind of mutual dependency ; and if they spring from the same cause or causes, circumstances may occasion the development of either symptom to the exclusion of the other. In the *fatal Typhoid* cases, tenderness was present in *four* cases, it was not present in *two*

cases, and its presence or absence was not stated in *one* case. It was present in each of the *three* fatal cases of *Typhus*. It was considerable in degree in two of the cases of *Typhoid*, moderate in one case, and the degree is not stated in the history of one case. It was moderate in degree in all the cases of *Typhus*.

*Gurgling*. By gurgling, I mean a *sound*, or a sensation appreciated by the *touch*, or both, when pressure is made over the abdomen, more especially in one or both of the iliac regions. Of the cases of *Typhoid*, this symptom was present in *twelve*, (*four* in the *private* and *eight* in the hospital cases.) Its absence was noted in *two* cases, and in the remainder, nothing is said on this point. In most of the latter it was undoubtedly absent at the time the daily examinations of records were made. Of the cases of *Typhus*, this symptom was present in *seven*, and in all the other cases nothing is recorded on the point. *Gurgling* was associated with *tenderness on pressure*, *tympanites*, and *diarrhœa*, as follows :—with *tenderness on pressure* in *nine* cases of *Typhoid* and in *two* cases of *Typhus ;* with *tympanites* in *eight* cases of *Typhoid*, and in *three* cases of *Typhus ;* with diarrhœa in *six* cases of *Typhoid*, and in *three* cases of *Typhus*. These enumerations suffice to show that there does not exist any uniformity of association of this symptom with the three symptoms just mentioned, either individually or collectively.

In the *fatal cases of Typhoid* nothing is stated relative to this symptom in *five ;* it was present in the *two* remaining cases. Of the *fatal cases of Typhus*, it was present in *one* case, and nothing is stated in the *two* remaining cases.

It will have been observed, that in the foregoing consideration of abdominal symptoms, the cases of *doubtful type* have been excluded. The facts with respect to the presence or absence of these symptoms in these cases, and the relations of the symptoms to each other, are exhibited in the following tabular arrangement of the *hospital cases of doubtful type :*

| | Case 1. | Case 2. | Case 3. | Case 4. [fatal.] | Case 5. | Case 6. | Case 7. | Case 8. |
|---|---|---|---|---|---|---|---|---|
| Tenderness. | absent | absent | present | present | absent | not stated | present | present. |
| Tympanites. | present | absent | present | absent | absent | present | present | not stated. |
| Gurgling. | present | present | present | present | not stated | not stated | not stated | not stated. |
| Costiveness. | present | not stated | present | present | present | not stated | not stated | not stated. |
| Diarrhœa. | absent | absent | absent | absent | absent | absent | absent | absent. |

### SECTION SIXTH.

*Cutaneous Eruptions.* The cutaneous eruptions incident to Continued Fever furnish, agreeably to the views of some distinguished observers, striking traits of distinction between the two types denominated *Typhus* and *Typhoid.* I shall, therefore, study the eruptions in the cases grouped after difference in type, separately, and compare the two types as respects the results. It should, however, be premised that in arranging the individual cases into the divisions of *Typhus* and *Typhoid,* considerable importance was attached to the characters, &c., of the eruption.

*Typhoid.* The characters, etc., which are said to distinguish what is called the *Typhoid eruption* are as follows:—It is generally limited to the chest and abdomen, but occasionally extends to the extremities. It may be copious, particularly over the chest and abdomen, but often it is the reverse, the spots being few in number. The eruption is of a rose red color, [rose spots, *taches roses,*] the spots are oval, appearing somewhat elevated, the redness momentarily disappearing on pressure.

In the histories of the *Typhoid* cases in which an eruption was present, the appearances, etc., were generally described, but in some instances, it is simply stated that it was marked by the *Typhoid* characteristics. I may fairly assume that in all cases in which variations from these characteristics were present they were noted, and that when the presence of the eruption is stated without special description, it exhibited the distinctive characters above mentioned.

A rose eruption was present in *nine* of the *twelve* cases in *private* practice, and in *fourteen* of the *eighteen hospital* cases, i. e., in *twenty-three* of *thirty* cases. In all the cases in which it was not stated as present, the fact of its absence was stated. It was present in a degree to be called copious in *five* cases in *private* practice, and in *two hospital* cases, i. e., in *seven* cases. In the other cases the number of spots varied from four or five to fifty. In one case only were they so few as four or five. In one case there were only five or six. In one case there were only ten. In all the others there were over fifteen. The eruption extended to the upper and lower extremities in *three private,* and in *one* of the hospital cases, in all *four.* It extended to the face in *one* of the *private* cases, and was associated with erythena of the face in that case.

The date of the development of the eruption can be ascertained in only a few of the cases. In many of the cases the disease had commenced several days before they came under observation, and the eruption was apparent from the first. In *six* cases in which the histories afford information on this point, the dates were respectively as follows:—In *one*, the *second* day after taking to the bed: In *one*, the *seventh* day: In *one*, the *fourth* day: In *one*, the *third* day: In *one*, the *fifth* day: In *one*, the *second* day. The *three* first of these cases occurred in *private* practice, the *three* last in *hospital*. These few cases suffice to show that the interval between the time that patients take to the bed, and the appearance of the eruption, is by no means uniform.

Data for determining the duration of the eruption are furnished in only *five* cases. In these cases it continued as follows:—In *one* case, *six* or *seven* days: In *one*, *twelve* days: In *one*, *eight* days: In *one*, *six* days, up to the day before the decease of the patient: In *one*, *ten* days. The duration, thus, in so far as these few observations enable us to judge, is far from being uniform. The only variations from the distinctive traits of the *Typhoid eruption*, which have been mentioned, were as follows:—In *one* case it was noted that in some of the spots the redness did not disappear on pressure. This was the only deviation from the typhoid characteristics in that case. In *one* case, some of the spots were vesicular, the contents of the vesicles being subsequently absorbed.

The size of the spots is not in the history of any case given. It is stated, however, in some of the cases, that the spots were of different sizes.

Of the *two fatal* cases of *Typhoid* in private practice, the eruption was present and copious in *one* case, and absent in the *other* case.

Of the *four* fatal *hospital Typhoid* cases, the eruption was present in *two*, and absent in *two*. It was not copious in either of the cases in which it was present. *Sudamina*, or miliary vesicles were not carefully looked for at the examinations of the patients. Their presence is only noted in a single case in the *Typhoid* group. In this case they were observed, on the fourteenth day, on the epigastrium, the patient having perspired profusely on that day. They were gone on the following day. It is very probable that they may have been present in other cases in which they were not noticed.

A *petechial* eruption was observed in *one* of the cases in the *Typhoid* group. The ecchymoses were not numerous, but sparingly scattered over the abdomen and lower extremities. The rose eruption was not present in this case. The case proved fatal, and the petechial spots were observed on the cadaver.

*Typhus*. The characters which are said to distinguish the *Typhus*

eruption are as follows:—The spots are smaller in size than in *Typhoid;* orbicular, not oval; not elevated; redness not disappearing readily on pressure; of a darker red or dusky hue. They are more frequently numerous, and extend oftener to the extremities.

Of the *thirteen* cases of *Typhus* in my collection, an eruption was present in every case. The eruption presented in all the distinguishing traits above mentioned, except that in *one* case some of the spots appeared slightly elevated. This case presented, thus, a combination of the eruptions of both types. The color was somewhat livid in *two* cases. It had a pink hue in *one* case. In *eleven* of the cases the eruption was copious. In *two* cases it was imperfectly developed. It extended over the extremities, more or less, in *eleven* cases. In the *two* cases in which it was imperfectly developed, the disease proved fatal, in *one* case, on the *fourth*, and in the *other* case on the *thirteenth* day. In the remaining fatal case in this group, the eruption was copious. The date of the development of the eruption, and its duration, are determinable in still fewer instances than in the *Typhoid* group. In *one* case it appeared the second day after the patient took to his bed. In another case it did not become developed until the *eighth* day. It continued six or seven days in *one* case, and five or six days in another case. In the histories of the remaining cases the data for determining these points are insufficient. A *petechial* eruption was present in *one* case, appearing on the *fourth* day after admission. The spots were observed on the lower extremities, and were of the size of the head of a pin. This patient had sloughing of the nates. The disease did not prove fatal.

*Sudamina* are not noted to have been present in any of the cases in the *Typhus* group. As remarked of the *Typhoid* cases, it is not improbable that they may have been present in some instances and escaped notice, since care was not taken to examine for this form of eruption.

On comparing the two groups as respects the foregoing results, it will be perceived that, with few and slight exceptional circumstances, the rules of discrimination which were enumerated, in so far as the present collection of cases is concerned, are sustained. I may add that this conclusion is unexpected, my impression having been that the exceptional circumstances would be found to be more numerous and striking.

It remains to submit the facts with regard to eruptions in the cases of *doubtful type.* Of the *eight hospital* cases of *doubtful type,* an eruption was present in *three.* In *one* of these cases *two* or *three rose-spots* only were observed. This was a fatal case. In *one* case the eruption was small and faint, and confined to the inferior part of the chest on either side, the redness disappearing on pressure. In the *third* case, the eruption appeared on the sixth day, and was copious, extending over the upper and lower extremities. It was of two kinds. The larger number of spots appeared somewhat elevated, and the redness disappeared on pressure. Other spots were not elevated, and the redness did not disappear on pressure. The eruption continued five or six days, gradually fading until it disappeared (which is the usual course with the eruptions in Continued Fever.) In the remaining *five* cases no eruption was present. In the *single* case of *doubtful type* in *private* practice, an eruption had existed prior to the case coming under my observation, but the characters were not ascertained. This was a fatal case. In the other fatal case two or three rose-spots were observed, and no autopsy was had in this case.

No inquiries occur to me suggesting comparisons of the eruption with other associated symptoms in individual cases.

SECTION SEVENTH.

*Symptoms referable to the Respiratory Apparatus. Cough. Expectoration. Pain in Chest. Pneumonitis. Aberrations of Respiratory Movements. Epistaxis. Singultus.*

*Cough.* More or less cough was present in *ten* cases of those in the *Typhoid* group, *five* being in *private* practice, and *five* in *hospital.* Of these *ten* cases, the cough was moderate, or slight, in *seven*, and a prominent symptom in *three.* It was present only during the early part of the febrile career in *three* cases. It was protracted into convalescence in *one* case. It occurred in connection with *pneumonitis* in *two* cases. In one of these cases the existence of that complication is demonstrated by recorded physical signs; in the other case physical signs are not stated, but the existence of the complication is evidenced by the rational symptoms.

Of the *Typhus* group more or less cough was present in *eleven* cases. Of these *eleven* cases, the cough was moderate, or slight, in *four*, and a prominent symptom in *seven.* It was present only during the early part of the febrile career, in a single case. It was protracted into convalescence in *two* cases. It occurred in connection with pneumonitis in *five* cases, in *three* of which that complication is determined by recorded physical signs; in the other *two* cases it is evidenced by rational symptoms.

The periods in the career of the fever of which the pneumonitis commenced in the cases of either type, are not given in the analysis preliminary to this Report, save in one instance, and the importance of determining this point is perhaps not sufficient to compensate for the trouble of re-perusing the histories of the cases in which this complication existed. In the case just excepted, the pneumonitis became developed twenty-seven days after the admission of the patient. Convalescence which was apparently postponed by the occurrence of this secondary affection, was not pronounced until the thirty-second day after his admission. The case referred to was in the *Typhoid* group.

It is not improbable that in some cases, in both groups, slight cough may have occasionally been present and escaped notice. In most of the cases in which the histories do not show that it was present, nothing is stated on the point, but it is not probable, and indeed hardly possible, that this omission could have occurred in any instance in which this was in any degree a prominent symptom. In *six* cases of *Typhoid*, and *six* of *Typhus*, more or less expectoration is stated to have accompanied the cough, but I am not sure that in all the other cases in which cough was present, it was wholly unattended by expectoration. In *two* cases of *Typhoid*, and *three* of *Typhus*, the expectoration is stated to have been muco-purulent. As respects the existence of pneumonitis, I am not satisfied that this complication did not exist, to some extent, in other cases than those in which it is now practicable to determine it to have been present. In general, physical exploration was not practised in cases in which the symptoms did not point to the existence of this complication; and it is certain that pneumonic inflammation may be present without being denoted by rational symptoms, even cough and expectoration being absent.

The foregoing results, however, suffice to show that cough and expectoration are symptoms incident to Continued Fever, without forming a necessary element of the disease; and that pneumonitis becomes developed in a certain ratio of cases, the average frequency of both, in so far as the present collection of cases is concerned, being indeterminate. But these results, imperfect as they may be, are interesting and striking, when considered and compared in the different types. It will be perceived that while cough was recorded present in only *one-third* of the cases of *Typhoid*, it was recorded present in *eleven-thirteenths* of the cases of *Typhus*. Admitting that these results are not perfectly exact, it is fair to conclude that it was present in a much larger proportion of cases of the latter, than of the former type. Moreover, it was a prominent symptom in only *one-fifth* of the cases of *Typhoid* in which it was present, and in more than *one-half* of the cases of *Typhus*. Pneumonitis was present in but *two* of the *thirty* cases of *Typhoid*, and in *five* of the *thirteen* cases of *Typhus*. These comparisons show a liability to pulmonary affections, considerably greater in the *Typhus* than in the *Typhoid* type of Continued Fever.

Of the *six fatal* cases of *Typhoid,* the presence of cough is not mentioned in the histories of *five,* and in the single remaining case, it was not a prominent symptom.

Of the *three fatal* cases of *Typhus,* it was present in all. It was a prominent symptom in only one case; it was present only in the commencement of the disease, in one case, and it was moderate in degree in the remaining case. It would thus seem that the presence of cough, and its prominency as a symptom, need not affect unfavorably the prognosis in either type of Continud Fever.

*Pain in the Chest.* This symptom is recorded present in but *one* case of *Typhoid,* and in this case it accompanied the development of pneumonitis on the twenty-seventh day after the admission of the patient. It was recorded present in *one* case of *Typhus,* occurring in connection with cough at the early pait of the febrile career, and situated beneath the sternum.

*Aberrations of Respiration.* Aberrations of respiration, more or less in degree, and differing in kind, appear in the histories of *nine* of the *thirty* cases of *Typhoid,* (*four* in *private* practice, and *five* in *hospital,*) and in *ten* of the *thirteen* cases of *Typhus,* thus showing a great preponderance in the latter type of the disease. In *eight* of the histories of the cases in the *Typhoid* group, it is recorded that the respiration was unaffected, and nothing is stated on this subject in the histories of *thirteen.* In the histories of the *three* cases of *Typhus* in which some aberrations of respiration were not noted, nothing is stated on the subject. The varieties of disorder in the respiratory movements, and the number of cases in which they were noted, are as follows:—*increased frequency* in *three* cases of *Typhoid,* and in *eight* cases of *Typhus; diminished frequency* in *three* cases of *Typhoid,* and in none of *Typhus; sighing respiration,* in *two* cases of *Typhoid,* and in *three* cases of *Typhus; panting on slight exertion* in *one* case of *Typhoid,* and *one* case of *Typhus*; *stertor* in *two* cases of *Typhus; catching* (inspiration shortened and quickened) in *one* case of *Typhoid,* and in *four* cases of *Typhus; irregularity in rhythm* in *three* cases of *Typhus; sibilant nasal rale,* in *four* cases of *Typhoid,* and in *two* cases of *Typhus; dilation of the alæ nasi,* in *one* case of *Typhoid* and in *four* cases of *Typhus.* These variations were, of course, more or less associated in different cases. It should also be stated that the aberrations of respiration occurring toward the close of life, in the cases which proved fatal, are not included, since they are, under such circumstances, incident to the mode of dying, and do not, strictly speaking, belong to the career of the disease.

It is of some interest to determine the relative frequency of these several kinds of aberration in cases of the two forms of Continued Fever; but a more interesting point of inquiry relates to their connection with the ulterior morbid conditions upon which they are dependent. Some of the symptoms that have been enumerated, are expressions of disorder of the nervous system, others are occasioned by the state of the pulmonary organs, and some may involve either or both. To bring the results of the analytical investigation to bear on this point of inquiry, a collection of cases might be divided into two groups, in the one group pulmonary disease being present as a complication of the fever, and in the other group this complication being absent. The two groups should then be studied and compared with respect to the presence of the several symptoms just mentioned. Symptoms present without the evidence, physical and rational, of pulmonary disease, it would be fair to refer to a nervous origin; on the other hand, those present in cases in which a pulmonary complication existed, might be due to it, or they might, still, originate in the nervous system; but if they exclusively existed in the group distinguished by the presence of pulmonary complication, they should be considered incidental to the latter. This interrogation of results is but imperfectly available in the present investigation, because, from neglect of physical explorations, it cannot be positively determined in what cases pneumonic complication was absent. I will, however, under distinct heads, institute, so far as practicable, a comparison in the manner pointed out, regretting that the data are not more satisfactory.

*Increased Frequency of Respiration.* In *four* of the cases of *Typhus* in which this variety of aberration was present, *pneumonitis* undoubtedly existed. Of the remaining *four* cases of *Typhus* characterized by this symptom, in all cough co-existed. In *one* case the cough was accompanied with sanguinolent expectoration. In another case the respirations were much accelerated (56 per minute the maximum) with labor, and panting on exertion. In this case pneumonitis may be suspected to exist. In another case, muco-purulent expectoration accompanied the cough, and occasionally the expectoration was streaked with blood. Dilation of the *alæ nasi* also co-existed. Either pneumonitis or bronchitis must have existed in this case as a complication. In the remaining case, the cough was a prominent symptom. Thus, *all* the *Typhus* cases attended with accelerated breathing, were complicated with pulmonary disease.

Of the *three* cases of *Typhoid* in which the respiratory movements were accelerated, the recorded data are quite insufficient for determining the presence or absence of pulmonary disease in one case. Nothing is stated with respect to cough. The respiration was catching, with dilation of the alæ nasi; coma and stertor succeeding. In another of the cases nothing is stated relative to cough. The respiration in this case was moderately accelerated, the maximum being 24. In the remaining case *pneumonitis* was evidenced by cough and rusty expectoration.

In conclusion, then, with respect to this symptom, in *all* but *two* of the *eleven* cases in which it was present in both types, it was accompanied by evidences of pulmonary complication; and in the two excepted cases the data are too defective for any definite inferences. I should state that in speaking of acceleration of the respiration as a symptom, I mean a degree of acceleration sufficient to attract attention. The histories of all the cases do not embrace an enumeration of the respiratory movements. When these did not appear to be increased they were not always enumerated. Hence, it may be that a slight increase above the average normal frequency may have existed in some instances when it was not sufficiently apparent to excite notice.

*Diminished Frequency of Respiration.* In none of the *three* cases of *Typhoid* in which this symptom was present, does the history afford evidence of pulmonary complication. In *one*, it is stated that no cough existed, and in the other *two* cases, nothing is recorded on that point. In the first case the respiration is noted to be somewhat slower and deeper than in health. Cerebral nervous symptoms, in this case, were not prominent. In one of the remaining cases cerebral symptoms were prominent, eventuating in coma. This case proved fatal. In the other case, the respiration was but slightly retarded, and more heavy than in health. In this case, about the average of cerebral symptoms were present. In so far as these few and imperfect observations go, diminished frequency of respiration is not to be attributed to morbid conditions of the lungs; and this accords with the view of this symptom generally entertained.

*Sighing.* By this I mean occasional, or frequent sighing, not a continuously suspirious respiration. Of the *two* cases of *Typhoid* in which this symptom was noticed, in *one*, cough was present at the early part, but in a trifling degree. In the other case cough did not exist. Of the *three* cases of *Typhus* in which the symptom was present, in *one*, cough and sanguinolent expectoration was present; in *one*, slight cough had existed, but did not continue up to the time that the case came under observation; and the remaining case was complicated with pneumonitis. If the character of this symptom be taken in connection with these results, it can hardly be doubted that it is due to a morbid condition of the nervous system. It is to be borne in mind that the co-existence of pulmonary disease in a certain proportion of cases, does by no means establish any connection between a symptom present in such cases, but also present in other cases in which pulmonary disease is absent. To prove such a connection the symptom should be present exclusively in cases characterized by pulmonary disease. I have been accustomed to regard frequent sighing as of bad omen in fever. This idea is, in some measure, sustained by the fact that in *two* of the *five* cases in which the symptom was noted, the disease proved fatal.

*Panting on slight exertion.* This was noted in *one* of the above cases of *Typhoid* in which *sighing* occurred, unconnected with any marked symptoms of pulmonary complication. In the other case cough and other pulmonary symptoms were prominent, and it is highly probable that pneumonitis existed.

*Stertor.* In both of the cases of Typhus in which this symptom occurred other than as an immediate forerunner of death, pulmonary symptoms were prominent, pneumonitis existing in one case, if not in the other. This, however, obviously, is not a symptom due directly to the condition of the lungs.

*Inspiration shortened and quickened.* In the single case of *Typhoid* in which this aberration is noted, the existence of secondary disease of the lungs is indeterminate from the history, no pulmonary symptoms being recorded, while cerebral symptoms were prominent. In the *four* cases of Typhus cough and other pulmonary symptoms were prominent, and in *three* of the cases pneumonitis was marked. In *two* of the *five* cases presenting this aberration, the disease proved fatal. This symptom I have been accustomed to regard as an expression of a morbid condition seated at the nervous centre, rather than in the pulmonary organs. The correctness of this view is not disproved, although it is in no wise sustained by the above results.

*Irregularity.* Of the *three* cases of *Typhus* in which this symptom was noted, in *one* case the history affords no evidences of the existence of pulmonary complication. In the *two* other cases pneumonitis coexisted. This, like the preceding aberration, is generally attributed to a nervous origin ; and the fact that, in one of the three cases in which it was present pulmonary disease did not co-exist, suffices to show that it is not necessarily dependent on the latter.

*Dilation of the alæ nasi.* In the single *Typhoid* case in which this was noted, the data are insufficient to determine either the presence or absence of pulmonary disease. In the *three Typhus* cases, cough existed in all. It was prominent in *one*, and the expectoration was streaked with blood; it was moderate in *one;* and in the *two* remaining cases, *pneumonitis* existed.

*Sibilant nasal rale.* This symptom has no special reference either to the nervous system, or pulmonary organs, but is probably dependent on the state of the nasal passages.

The foregoing examination of cases with a view to determine the connection subsisting between the several aberrations of the respiratory movements, and the secondary affections of the pulmonary organs occurring in Continued Fever, may not seem to furnish results sufficiently numerous and explicit to compensate for the labor which it has cost. I should perhaps hardly deem them deserving of the space which they occupy in the report, were it not that, possibly, they may serve to suggest the application of a similar method of comparison in a larger series of cases with the facts relating to the pulmonary system more satisfactorily recorded.

The reader may be surprised that in connection with the subjects embraced in this Section, I do not adduce the appearances presented on dissection of the pulmonary organs, in the instances in which the disease proved fatal. The reason for this omission will appear in the Section devoted to the Autopsical Observations, where it will be perceived that the post obit histories of the fatal cases are very imperfect as respects the pulmonary organs.

*Epistaxis.* Epistaxis was noted in *eight* of the cases of *Typhoid* (*three* in *private* practice and *five* in *hospital*). In *three* cases it is noted that this symptom did not occur. In the remainder nothing is said either of its absence or presence. In all the above eight cases the hemorrhage occurred from the nostrils, more or less in quantity. In a few cases (*three*) not included in this enumeration, it was observed that sputa detached from the posterior nares were tinged with blood. This may have occurred in other instances and have escaped observation. It is also not impossible that epistaxis may have occurred in some of the cases not under observation from the commencement of the disease, the fact not being ascertained, although, generally, if not universally, pains were taken to inquire whether this symptom entered into the previous history. In most of the cases in which nothing is stated relative to this point in the history, it is fair to presume the symptom did not occur. Here, as in other instances, the importance of recording negative facts was not sufficiently borne in mind. As respects the period in the career of the Fever when epistaxis was observed, in *all* but *three* instances this is stated in the preliminary analysis. In these three cases it is simply stated that it occurred early. In the other cases the periods were respectively as follows :—In *one* case, on the 8th and 10th days; in *one*, on the 8th day; in *one*, daily, up to the 7th day, and sometimes profusely; in *one*, on the 10th day; and in *one*, on the 26th day. The ratio in which this symptom was observed in many cases is very nearly the same as in the 303 cases of Typhoid Fever analyzed by Dr. Jackson. Of the quantity of blood which escaped, and the precise number of times the hemorrhage took place, the histories do not contain information. Pains were not taken to ascertain the former more especially. Generally speaking, it was slight, and I am quite certain that no appreciable effect upon the progress of the disease was produced by it. In none of the cases in which epistaxis occurred, did the disease prove fatal; and *two* of the *three* cases in which it was noted that this symptom did not occur, were fatal cases. Of the cases of the *Typhus* group, epistaxis was noted in but *two*. In *one* of these it occurred several times early in the febrile career; in the other case it occurred once early, and once afterward. In one of these cases the disease proved fatal. In none of the cases of Typhus is it noted that the sputa from the posterior nares were tinged with blood. In *three* of the cases in this group the absence of this symptom is noted. In the other cases nothing is stated on the subject. Comparing the foregoing results in the two groups, this symptom occurs oftener in *Typhoid* than in *Typhus*, the ratio being a little more than one in four of the former, and one in six and a half in the latter type.

*Singultus.* This symptom was observed in *two* cases, *one* of *Typhoid*, and the *other* of *doubtful* type. In the former case it was a troublesome symptom for several days. The disease proved fatal, but this symptom ceased toward the close of life. It was unaccompanied by involuntary movements of the voluntary muscles. In the other case, it occurred on the day after the admission of the patient, four grains of opium having been administered the night previous. The respiration was, at the same time, somewhat labored, and the inspiration shortened and quickened. This case became complicated with pneumonitis.

In the foregoing enumerations and comparisons with respect to the symptoms referable to the respiratory system, it will here be observed that the cases of *doubtful* type (with the single exception in the preceding paragraph) have not been included. It is probably not of much importance to present the facts in the histories of these cases belonging to this class, but since it has been done hitherto, it will render the report defective to omit them, and as the readiest mode of accomplishing the object they are arranged in the following tabular form:—

